# Efficient Mako Shark-Inspired Aerodynamic Design for Concept Car Bodies in Underground Road Tunnel Conditions

**DOI:** 10.3390/biomimetics9080448

**Published:** 2024-07-24

**Authors:** Ignacio Venegas, Angelo Oñate, Fabián G. Pierart, Marian Valenzuela, Sunny Narayan, Víctor Tuninetti

**Affiliations:** 1 Department of Mechanical Engineering, College of Engineering, Universidad del Bío-Bío, 4051381 Collao Avenue, Concepción 1202, Chilefpierart@ubiobio.cl (F.G.P.); 2 Department of Materials Engineering (DIMAT), Faculty of Engineering, Universidad de Concepción, Concepción 4070409, Chile; 3 Doctoral Program in Sciences of Natural Resources, Universidad de La Frontera, Casilla 54-D, Temuco 4780000, Chile; m.valenzuela16@ufromail.cl; 4 Department of Mechanics and Advanced Materials, Campus Monterrey, School of Engineering and Sciences, Tecnológico de Monterrey, Av. Eugenio Garza Sada 2501 Sur, Tecnológico, Monterrey 64849, Nuevo León, Mexico; s.narayan@tec.mx; 5 Department of Mechanical Engineering, Universidad de La Frontera, Temuco 4780000, Chile

**Keywords:** biomimetics, computational fluid dynamics, numerical simulation, bionic car design, nature inspiration, underground tunnels, drag reduction

## Abstract

The automotive industry continuously enhances vehicle design to meet the growing demand for more efficient vehicles. Computational design and numerical simulation are essential tools for developing concept cars with lower carbon emissions and reduced costs. Underground roads are proposed as an attractive alternative for reducing surface congestion, improving traffic flow, reducing travel times and minimizing noise pollution in urban areas, creating a quieter and more livable environment for residents. In this context, a concept car body design for underground tunnels was proposed, inspired by the mako shark shape due to its exceptional operational kinetic qualities. The proposed biomimetic-based method using computational fluid dynamics for engineering design includes an iterative process and car body optimization in terms of lift and drag performance. A mesh sensitivity and convergence analysis was performed in order to ensure the reliability of numerical results. The unique surface shape of the shark enabled remarkable aerodynamic performance for the concept car, achieving a drag coefficient value of 0.28. The addition of an aerodynamic diffuser improved downforce by reducing 58% of the lift coefficient to a final value of 0.02. Benchmark validation was carried out using reported results from sources available in the literature. The proposed biomimetic design process based on computational fluid modeling reduces the time and resources required to create new concept car models. This approach helps to achieve efficient automotive solutions with low aerodynamic drag for a low-carbon future.

## 1. Introduction

All living organisms possess traits derived from the natural world that facilitate their adaptation to specific ecological niches [1]. These attributes help species to survive under challenging environmental circumstances by allowing them to evade predators. Numerous types of plants and animals have evolved distinct physiological characteristics that pique human interest. These include, for instance, the scales on the shark’s body, which reduce hydraulic drag [2]. These biological features can be transformed into technical solutions to aid in the resolution of current engineering problems using “biomimetics”. This branch of science deals with the study of natural biological features for the benefit of humanity [3]. The Airbus Bird of Prey project is one such example [4]. The eagle’s wing and tail features were mimicked to provide active flight control. Biomimetic fish scale arrays (FSAs) also showed great potential as a biomimetic solution to reducing drag [5,6]. The “Shinkansen E5” bullet train managed to reach a speed of 320 km per hour, overcoming the problem of compliance with Japan’s noise pollution standards [7] by observing the features of a kingfisher bird quickly diving into water to capture prey with minimal noise and disturbance. Other biomimetic case design is the Eastgate (Zimbabwe) design inspired by the energy-efficient mounds built by African ants [8]. These termite mounds have the ability of maintaining constant temperature and humidity, despite outside temperature variations ranging from 2 to 42 °C. The constant humidity levels cause fungi to grow inside. These mounds are like towers with many openings on their sides, allowing cold air entry. Thus, when they come in contact with the lower-temperature air inside, they generate a heat exchange via convection, causing natural fluid circulation. Speedo is a clothing brand for aquatic athletes that has been innovative in creating swimsuits inspired by a shark’s qualities [9]. The texture and orientation of the animal’s skin considerably reduces the friction and turbulence caused when moving through the water. This is how Speedo created the “Fastskin” suit, which moved away from traditional models, considerably reducing water’s resistance on a swimmer’s surface. In addition to generating efficiency when swimming, this decreased the amount of water absorbed and the vibrations generated. This reduction gives swimmers greater oxygen flow to the muscles, which means they can perform laps at a greater speed [10].

As the automotive industry is driven by innovative solutions, there is a demand to apply biomimetic concepts for design strategies to solve problems faced by engineers [11]. This includes the application of a multi-domain approach to effectively achieve new car designs with improved performance.

Since the early days of the automotive industry, designers have sought to incorporate the aerodynamic shapes of birds and fish into vehicle design to improve both performance and aesthetics. Bioinspired vehicle designs in the automotive field include the boxfish [12], tiger beetle [13], tiger and cobra [1]. In the same context, Mercedes-Benz has developed a vehicle called the bionic car, inspired by the boxfish [12]. The car demonstrates an extremely low drag coefficient of 0.19, compared with the typical range of 0.30–0.35, resulting in 35% fuel savings. Reductions in aerodynamic drag of 20% have also been reached for trucks, such as the cabin roof fairing inspired by the front body of the sea lion [14]. In buses, biomimetic optimizations based on belugas achieved a 12.6% reduction in fuel consumption [15]. Bioinspired accessories also improve vehicle performance. For example, the tubercle effect in whales and a rear spoiler on a vehicle have been found to enhance efficiency [16,17]. In one case, optimizing only the cabin of a light truck based on the yellow boxfish’s head led to a 30% reduction in aerodynamic resistance compared with that of the commercial system [18].

Since large cities are currently facing problems of vehicle oversaturation and heavy traffic, the use of underground tunnels as a measure to mitigate traffic congestion has recently received attention. This approach serves as a structural or engineering solution to effectively relieve pressure on surface roads [19,20] by providing additional routes for vehicle flow [21,22]. However, these proposals face significant constraints, such as high construction and maintenance costs, as well as the potential environmental and social impacts arising from the excavation and use of tunnels [22]. Highway construction in mountainous regions, where the length of tunnels varies according to geological conditions, technical requirements and budgetary constraints, has been a major focus in the application of these engineering systems [22].

In China, tunnel construction has been actively promoted since 2022, with more than 23,200 structures completed to date [23]. The efficient design of these structures is crucial to alleviating traffic congestion; otherwise, other significant traffic problems may arise, especially around tunnel entrances, which are responsible for 20% of congestion [24]. Fang et al. [25] investigated the tunnel length impact on traffic congestion. Additionally, Yeung et al. analyzed traffic accidents in highway tunnels, finding higher accident rates in transition zones compared with interior zones. Overall, highway tunnels were generally safer with reduced traffic capacity [26].

The main objective of this study is to explore the potential application of shark-biomimetic designs in automotive use within underground road tunnel conditions. Underground road tunnels for vehicles can provide a solution to surface congestion, resulting in smoother traffic flows and shorter travelling times. This, in turn, has the potential to reduce urban noise pollution, creating a more peaceful and desirable living environment for city dwellers. To achieve aerodynamic efficiency, a selection process was conducted to find the body surface profile inspired by the mako shark. The influence of the bioinspired design on the vehicle’s aerodynamic resistance was evaluated through an adjustment to the mako shark’s morphology and subsequent parametric optimization. This aquatic animal is the fastest in the marine world, easily reaching speeds close to 74 km/h due to its efficient body shape, which enables it to generate low resistance to movement [27]. As a result, it is known as the “peregrine falcon of the sea”. The geometry of the shark tip appears to be very attractive for achieving the aerodynamic efficiency parameters sought. This approach has not been found in automotive applications, although shark denticle-inspired designs for improving the aerodynamics of airfoils have been studied previously [25], showing significant results in terms of an increased lift-to-drag ratio [28].

## 2. Adaptive Bioinspired Applications in Automotive Technology

**Figure 1:**
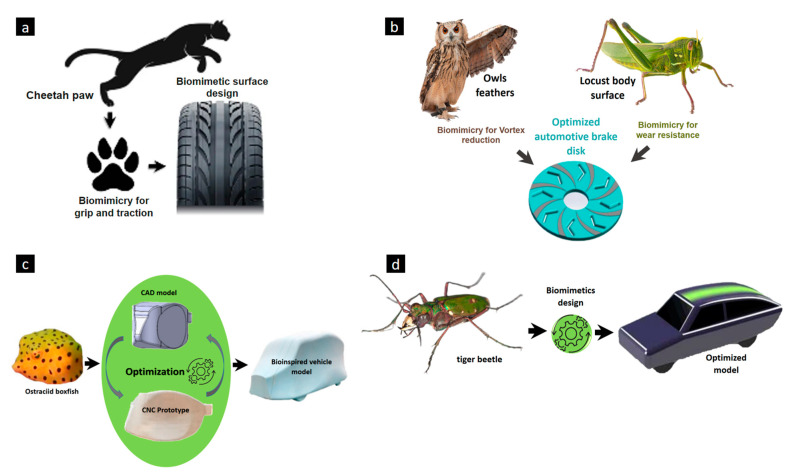
Biomimetic designs. (**a**) Surface of tires based on morphology of cheetah’s paws. (**b**) Bioinspired automotive brake disk. (**c**) Boxfish-inspired car. (**d**) Tiger beetle-inspired bionic car.

Automotive engineers need to formulate strategies for the key issues of smart and sustainable futuristic transportation. Exterior aesthetics are of prime importance in the future’s individualistic social scenario in which bioinspiration will play a prime role in satisfying the consumer. A whale’s biological features and mechanisms were incorporated into the aesthetic design of the proposed futuristic vehicle [29]. The automobile manufacturer Nissan introduced self-cleaning in Nissan Note based on the super hydrophobic effect [30]. Bioinspired polypropylene panels inspired by the carapace structure of the red-eared slider and Harakeke were designed for the automotive interior [31]. In terms of functional efficiency in vehicles, the honeycomb structure for the design of diesel particulate filters [32] and improved properties of materials parts [33] based on biomimetic were identified. Ganilova et al. [34] designed a honeycomb-inspired structure for energy absorption for an automobile bumper. Ivanović et al. [35] adopted the cheetah paw’s structure in tire design to improve grip by reducing road friction (Figure 1a). A brake disc with reduced noise and better wear resistance has been designed in accordance with the layout of the owl’s feathers and the surface of the locust (Figure 1b) [36]. Particularly, in terms of aerodynamic improvements in vehicles and efficiency, the Mercedes-Benz company has introduced a bionic car inspired by a boxfish (Figure 1c) [12]. Peng et al. [13] designed a low-drag car inspired by the shape of a tiger beetle, as seen in Figure 1d. The introduction of dimples on the hood surface was reported to have resulted in a significant reduction in the drag coefficient (Cd) [37]. Kim et al. [38] explained the working of an automatic moving deflector (AMD) inspired by the design of the feathers of a bird to reduce air drag. These proposed systems with good energy performance features comply with the global sustainable targets of improving systems’ efficiency and reducing carbon emissions [39].

The application of biomimetic principles in the automotive industry has demonstrated beneficial results. However, despite the potential of bioinspired vehicle design to improve aerodynamics, it is necessary to continue to explore new opportunities for improvement based on evolutionary advances that will further enhance vehicle performance and aerodynamic efficiency. Consequently, the identified research gap can be filled with new conceptual vehicle designs for underground tunnel automotive applications based on animal shapes with high kinematic performance.

## 3. Bioinspired Design Process

**Figure 2:**
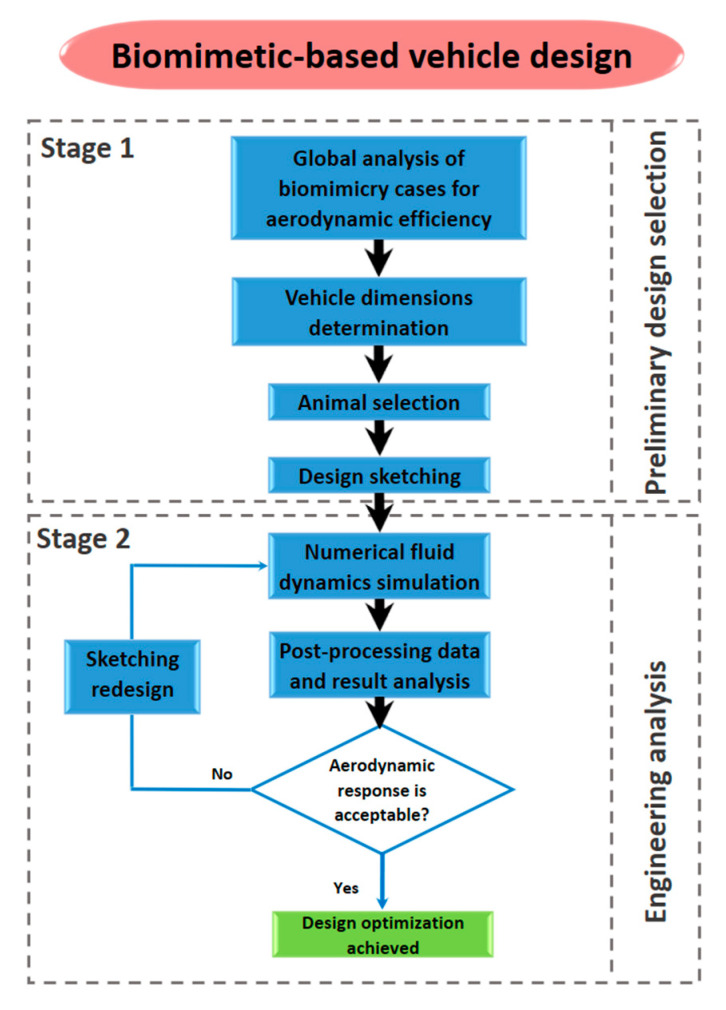
Proposed biomimetic-based design method using computational fluid dynamics.

The different steps of the biomimetic-based design process proposed in this study for mako shark-inspired vehicle design is globally visualized in the diagram of Figure 2. The first stage of preliminary design selection includes a global analysis of biomimicry cases and animals with high performance kinetics and dynamic flow. The next step is to define the specific application constrains such as the design space dimensions of vehicles. The initial phase concludes with the creation of design sketches, which involves incorporating vehicle design limitations with the shape of the animal’s body selected as the most suitable and performant for the application. The engineering analysis in the second stage follows an iterative design process based on computational fluid dynamics (CFDs) until the required aerodynamic behavior is reached. More details of the procedure are described hereafter.

### 3.1. Shark Body Profiling in a Restricted Vehicle Design Space

**Figure 3:**
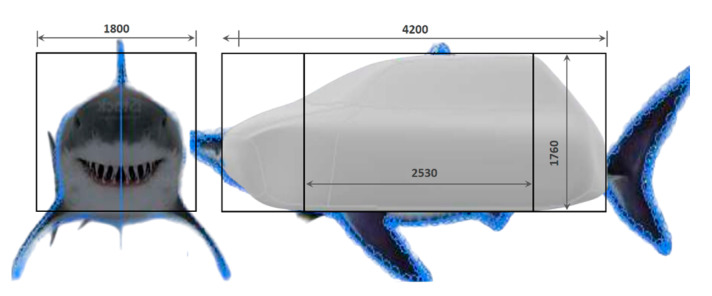
Spatial dimensions for bionic vehicle designs.

**Figure 4:**
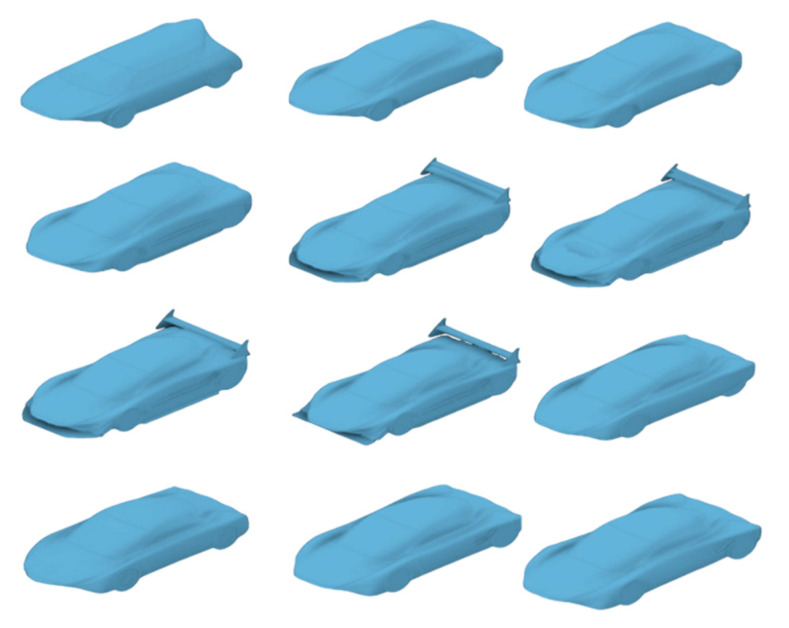
Compilation of shapes from the convergence process.

Designing an object based on an animal requires careful consideration, as it is impossible for the final product to possess all of the animal’s qualities or shape features. Different shapes should be created, modified and optimized, taking into account several factors. These factors include aesthetics and ergonomics suitable for the application and user, dimensions appropriate for on-road conditions and aerodynamic performance for fuel efficiency. An initial sketch was made based on the profile and frontal view of the shark (Figure 3) and design space constraints. Three-dimensional modeling with sequential parametric optimization was carried out in Autodesk Inventor 2022 and Ansys Workbench 2022 R2 software. Standard commercial vehicle dimensions were used to adjust the vehicle and ensure that the design and simulation were representative and adequate for the application (Figure 4).

### 3.2. Shape Design Convergence of First Prototype

**Figure 5:**
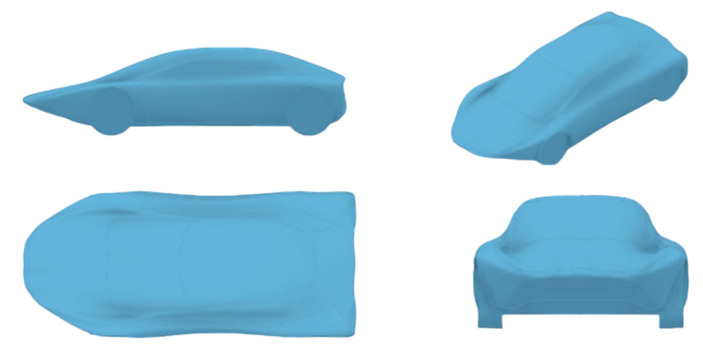
Shape of prototype.

Creating a vehicle based on an animal’s geometry is a process that requires numerous sketches and the subsequent simulation of each one. The animal’s profile was adapted to the vehicle design space for a complete representation of initial shape design. Then, the optimization process of the car was based on controlling aerodynamic parameters to achieve an efficient response. Several iterations for the convergence of the selected turbulence model of air flow were performed to ensure the reliability and quality of results. Once the initial vehicle’s aerodynamic parameters were calculated, sequential parametric design optimization included shape modifications and aerodynamic elements such as front and rear spoilers to reduce aerodynamic effects such as drag and lift coefficients, while maintaining control over the aerodynamic forces of the system. To create a prototype with low and acceptable aerodynamic drag and an optimal lift coefficient, over 30 different models were developed. The shapes of the models were varied in accordance with the particular pattern of flow entry at the front of the vehicle and the slope of the rear where the fluid separates from the surface. This controlled the pressure profile, aerodynamic forces, and drag and lift coefficients. Similar to the shape profile of the mako shark, the end area of the vehicle must be adjusted to a smaller geometry to reduce the drag generated by pressure drops and turbulent airflow behavior. The area of the tire surfaces interacting with the air at the front of the vehicle also received attention in the design. Some curves were modified to achieve harmonious airflow behavior. The pressure and velocity contour on the vehicle’s profile were analyzed in each case with drag and lift coefficients. A selection of different prototypes simulated during the iterative process are given in Figure 4, while Figure 5 shows the selected adequate design at this process stage.

### 3.3. Numerical Simulations of Air Flow Dynamics

**Figure 6:**
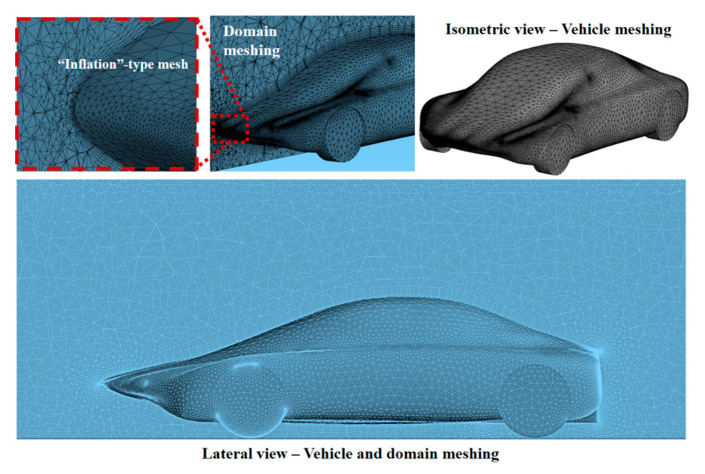
Meshing of the domain in section view and the vehicle in isometric view, including the inflation type on the vehicle surface.

**Figure 7:**
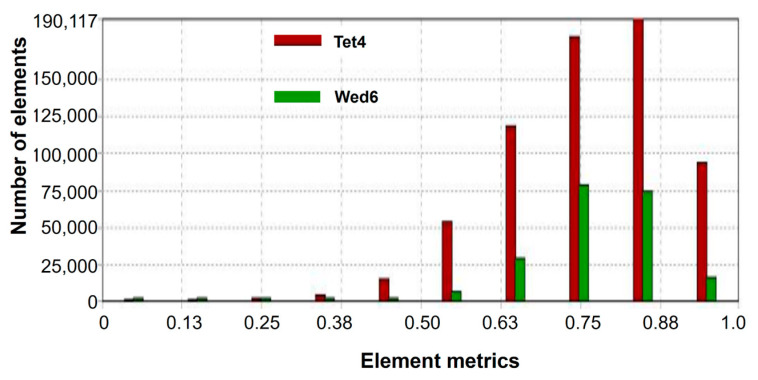
Mesh quality graph.

The numerical simulation of aerodynamic behavior was performed using ANSYS 2022 R2 software. The meshing stage is fundamental as it directly influences convergence, simulation speed and the accuracy of the results. The default mesh configuration was modified with the inflation method to generate small adjacent cells on the vehicle surface contour and boundary layer to better capture aerodynamic performance and result variables (Figure 6). Tetrahedral (Tet4) elements were selected as they offer flexibility in achieving a high-quality mesh for the investigated configuration. Prismatic elements (Wed6) were included to improve geometrical discretization and convergence (Figure 7). The patch-conforming method was chosen due to the absence of any geometry issues such as dirt or poor quality.

The dimension of each element was 200 mm. By default, the program delivers an element size of 700 mm, modified to be more precise in the results. From the outline of the profile, the geometry of the elements varies; this is generated by the “inflation” command. The total number of cells for the full model with acceptable results was about 0.85 million.

**Figure 8:**
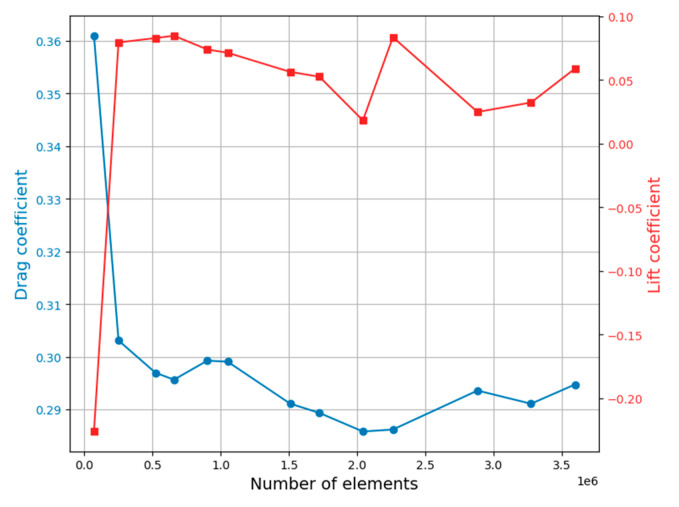
Mesh convergence study.

The mesh convergence study exhibited a variation of less than 6% for the grid resolution of 2.5 × 10^5^ elements (Figure 8). The orthogonal quality metric selected to measure the mesh quality exhibited a larger number of elements between 0.63 and 1.00. This range classified the mesh as being of very good quality for the simulation. The reader is referred to Buscariolo et al. [40] for mesh analysis cases of the well-known Ahmed body model.

#### 3.3.1. Model Configuration

The fluid configuration across the domain was carried out using the K-epsilon model due to its extensive applications in the automotive industry [41,42]. The constants used in the model were the turbulence quantity, K, a C_µ_ value of 0.09, a C_ε1_ value of 1.44 and a C_ε2_ value of 1.92. Values for the variables K and epsilon were utilized within the range of 0.001 to 0.015 to avoid the predominance of the initial flow state.

#### 3.3.2. Boundary Conditions

**Figure 9:**
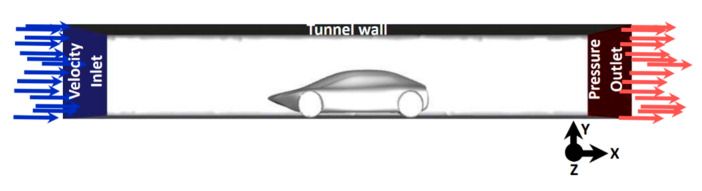
Domain configuration.

The conditions of each domain surface must be established to obtain adequate results. The entry condition is defined as the velocity inlet boundary: air flow at atmospheric pressure with a uniform velocity of 100 km/h and 5% turbulence intensity. The ground must also have displacement in the same flow direction as that of the entry surface to simulate vehicle movement. The car was established as a stationary wall, with an angular rotation speed of 85.2 rad/s assigned for wheels and zero pressure at the domain outlet (Figure 9). The dimensions of the flow domain simulating the passage through the underground road tunnel are as follows: width 3.92 m, height 2.18 m and length 14.34 m. The no-slip surface condition was defined.

#### 3.3.3. Drag and Aerodynamic Efficiency Control

The key indicators of the aerodynamic efficiency of the designs are the obtained values of the drag (Cd) and lift (Cl) coefficients. For this purpose, a projection of the frontal area of the vehicle was required. The lift and drag forces were decomposed to determine the coefficients using well-known drag and lift equations. Thus, after completing the simulation with converged results and low residuals, values of the drag and lift coefficients were calculated. For optimizing the vehicle designs, velocity and pressure contours as well as streamline airflows were also computed and controlled.

## 4. Results

### 4.1. Analysis of Convergence and Reliability of Numerical Results

**Figure 10:**
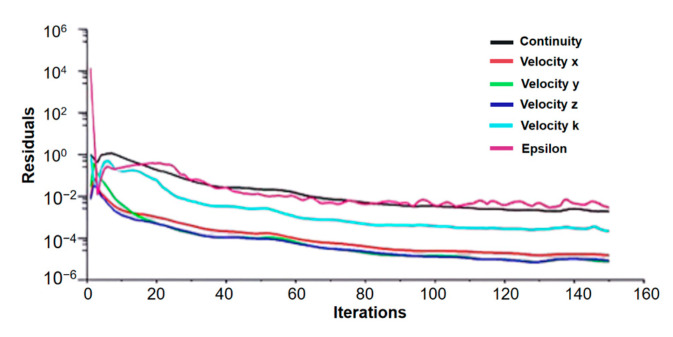
Residual values vs. number of iterations.

To provide reliable and stable results, a convergence analysis of lift and drag coefficients, including other variables such us velocity and pressure, was computed. The scalar residuals as a function of the number of iterations defines the behavior of the variables in the iterative process (Figure 10). The 150 iterations showed good convergence results for velocity, continuity and the k-value.

**Figure 11:**
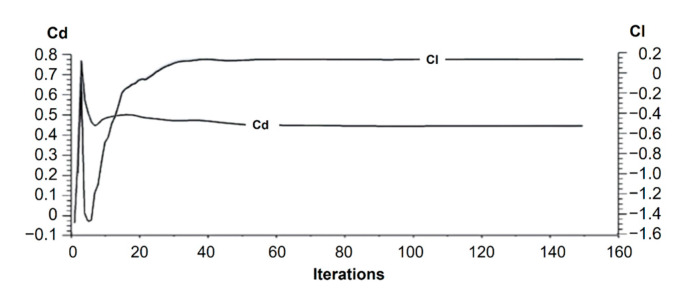
Cd and Cl vs. number of iterations.

The convergence analysis of drag and lift coefficients is shown in Figure 11 to ensure the reliability of numerical results. Note that relatively good convergence is reached after about sixty iterations. However, residuals of the velocity vector require at least 120 iterations for adequate results (Figure 10).

The drag coefficient (Figure 11) showed significant variation in the first 10 iterations, highlighting the importance of conducting a high number of iterations to ensure result convergence. Beyond 60 iterations, the behavior becomes more stable and yields a Cd value of 0.44. The exhibited high drag force of 439 N indicates that the further optimization of the car’s shape is required to reduce inefficient aerodynamic behavior.

The Holden car has been reported to have a Cd value of 0.4 at 100 km/h, and a value of 0.44 with a taxi sign [43], while boxfish-inspired cars reached a Cd value of 0.24 [12]. At this early stage, vehicle design model 1 with a high Cd value clearly cannot be considered finished yet, even when compared to traditional commercial vehicles without biomimetic optimization. The next section shows improvements being made in this model by applying the iterative process of mako shark biomimetic vehicle design with full-field computational fluid dynamic modeling.

**Figure 12:**
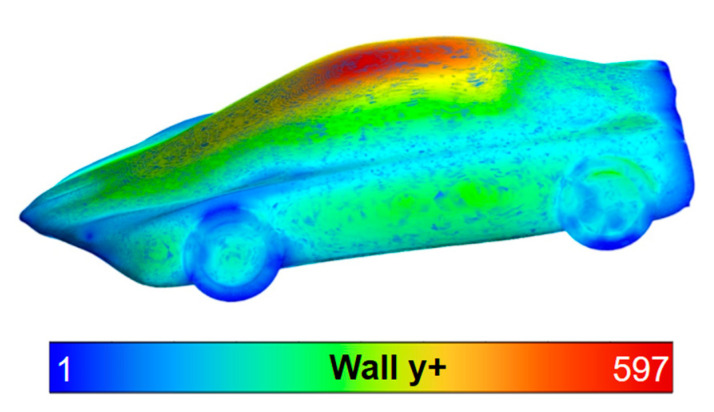
Design 1 showing y+.

The quality of the results and the correct adaptation of the mesh were also confirmed using the y+ parameter, thus verifying that the dimensional distance between the wall and the first cell of the mesh near the wall allows for the proper modeling of the near-wall boundary layer behavior. In this manner, we accurately modeled the viscous effects experienced in the simulation of underground tunnels due to wall interaction. For this purpose, the mean value of y+ should be in the range of 30 to 400 to ensure adequate boundary layer resolution in turbulence boundary layer models (k-epsilon). The mean value of y+ obtained is 318.2, reaching maximum values of 597 in the upper zone of the vehicle. Based on the above, it is confirmed that the boundary layer resolution is adequate (Figure 12).

The values of the forces are very important for defining the vehicle purpose. The vehicle obtained a Cl value equal to 0.155 and a lifting force of 585.6 (N). These values are very high compared to with the ranges of street vehicles reported [44]. The Formula One car clearly has a greater load due to its geometry and configuration of aerodynamic elements, such as spoilers, a flat bottom and diffusers [45]. This allows the vehicle to take curves at a high speed with more grip. On the other hand, the lift force on a street vehicle traveling at 100 km/h should be close to 0. This emphasizes the need for design optimization to minimize lift parameters, ensuring that the vehicle is tailored to its intended operating conditions.

**Figure 13:**
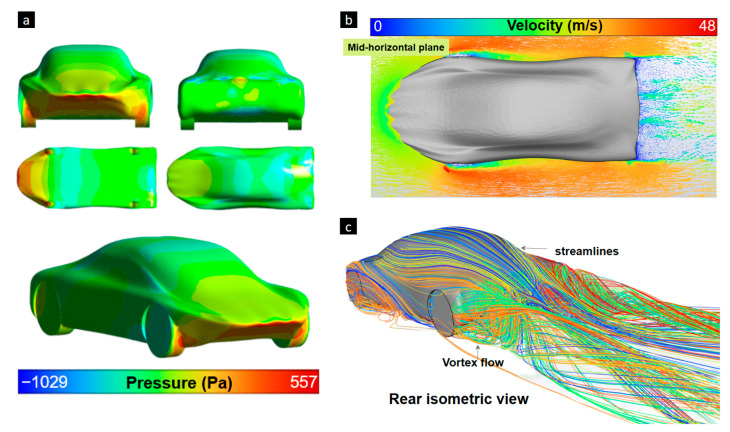
Design 1 showing (**a**) the pressure contour in different views, (**b**) air velocity in the horizontal mid-plane and (**c**) streamlines of air surrounding the vehicle surface.

The interaction of air with a vehicle’s surfaces is analyzed for making modifications to minimize wind-induced forces. Analyzing turbulence, flow speed variations, and pressure differences helps in studying and enhancing the design. Once the required pressure and speed parameters are identified, a comprehensive assessment can provide aerodynamic improvements. The captured surface pressure contour of Design 1 is presented in Figure 13a. High-pressure areas can be seen at the front and in the tires because these areas impact the fluid. Pressure variation is displayed in other areas, such as at the top and bottom. Pressure traces can provide valuable information about the extent of laminar flow and the amount of lift generated by the body–air interaction, and can also indicate potential areas of flow separation. Figure 13b,c provide additional data for the aerodynamic performance assessment of the design. Note that the non-perfectly symmetrical results obtained in the downstream are as expected due to vortex and numerically sensitive instabilities. Even if symmetrical models are designed, the automatic mesh does not generate perfectly symmetrical spatial discretization. Figure 13c showing the streamlines’ flow is included to support the resulting static pressure and velocity contour plots. Similar asymmetrical results of the velocity field were obtained in other related research by Zhang et al. [46].

### 4.2. Optimizing the Aerodynamic Performance of the Mako Shark-Inspired Concept Car

The iterative design process sought to reduce aerodynamic effects such as drag and lift coefficients while maintaining control over the aerodynamic forces of the system. To create a prototype with low aerodynamic drag and an optimal lift coefficient, over 30 different models were developed to reduce the coefficient to acceptable values. These models varied in the singularity of flow entry at the vehicle’s front and the slope of the rear where the fluid separates from the surface. This controlled the pressure profile, aerodynamic forces, and drag and lift coefficients. Like the mako shark profile, the final section must have a smaller geometry to reduce the drag generated by pressure drops and turbulent airflow behavior.

**Figure 14:**
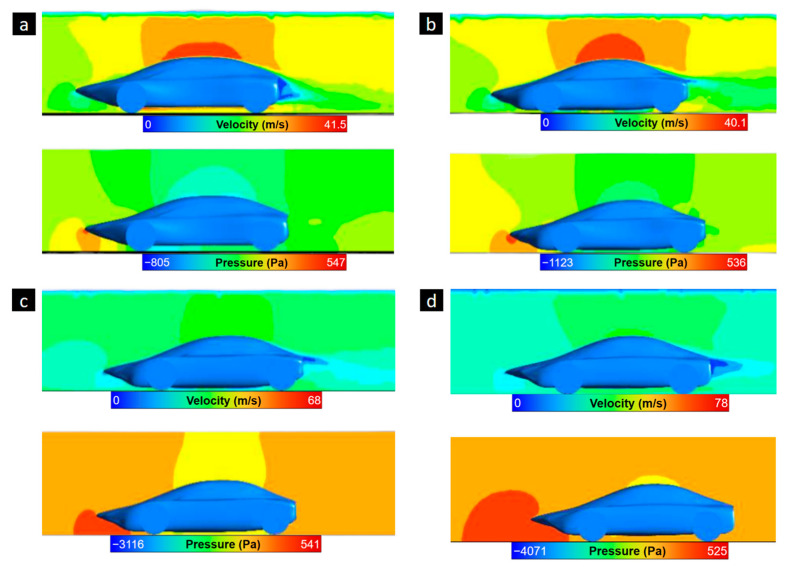
Numerical results of velocity and pressure contour in the vertical midplane for (**a**) Design 1, (**b**) Design 2, (**c**) Design 3 and (**d**) Design 4.

At the front, the surface of the tires impacting the air must be covered, and certain curves must be modified to ensure the fluid has harmonious behavior upon impact with the vehicle’s frontal area. A middle vertical plane was generated and configured to capture a pressure and velocity contour on the vehicle’s profile. Air flow velocity and pressure fields are collected and shown for Design 1 to Design 4 (Figure 14). Improvements in the front and rear could be observed until reaching a final design, which will be analyzed for further optimizations. The initial designs of the model did not include an air diffuser, and the slopes in the flow inlet and outlet were not smooth enough, resulting in unsafe aerodynamic forces for a functional prototype. Due to this reason, optimizations consisted of incorporating aerodynamic components to optimize the forces interacting on the vehicle and the drag and lift coefficients.

Design improvements were developed by analyzing the pressure and velocity plane, which allowed a visualization of the areas where air moved in a laminar or turbulent manner, as well as the velocity the flow maintained in specific areas. The distribution fields (Figure 14) illustrate how altering the curve of the vehicle’s roof influences the aerodynamic forces generated. Although there was a slight geometry change at the front, the effect of the profile of the mako shark in the vehicle design is still visible. Finally, Design 4 achieved the best aerodynamic performance in terms of drag and lift coefficients. The values obtained are a Cd of 0.28 and a Cl of 0.05. This optimization was performed because the first design was not aerodynamically efficient. This design differs slightly from one of the most efficient biomimetic designs in the automotive field, the boxfish, with a Cd value of 0.24 [43].

### 4.3. Diffusers for Improving Aerodynamic Efficiency

**Figure 15:**
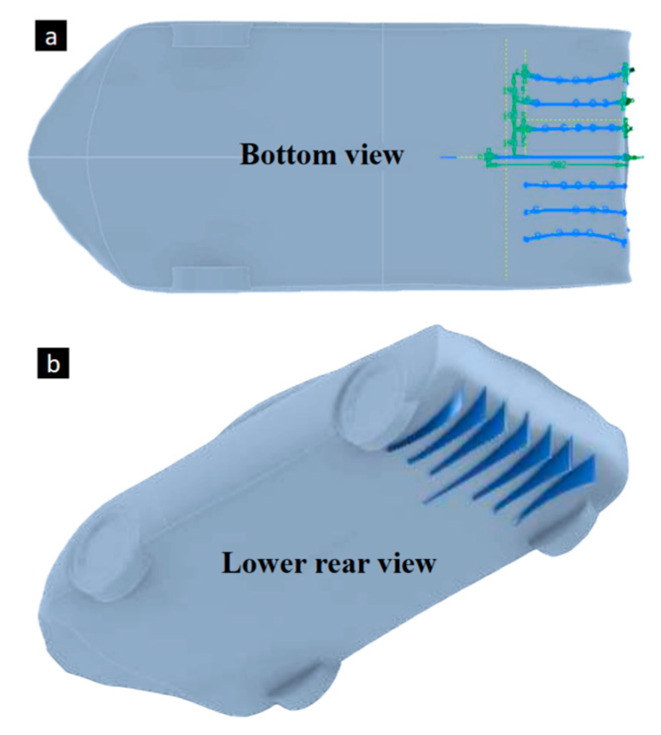
Diffuser design for downforce increase.

The iterative design process is essential when seeking the best results of efficiency. Design 4 with the best aerodynamic performance was evaluated for the implementation of the aerodynamic diffuser in the lower rear area. These devices are known to organize the airflow, mitigating turbulence. The criteria for reducing the lift coefficient by increasing the velocity of the air passing underneath suggest that this is the appropriate approach. After trying various diffuser designs, the one shown in Figure 15a was chosen. This design includes a curvature in the components that face the wheel of the vehicle to divert the air to areas where it does not reach, such as in the rear area of the rear tire. Once the profiles were modeled, the extrusion of the area of the diffuser components was carried out considering the adequate distance from the ground (Figure 15b).

The simulation of Design 4 with a diffuser was performed to evaluate the behavior and visualize the pressure differences under the vehicle. The parameters obtained are shown in Table 1.

**Figure 16:**
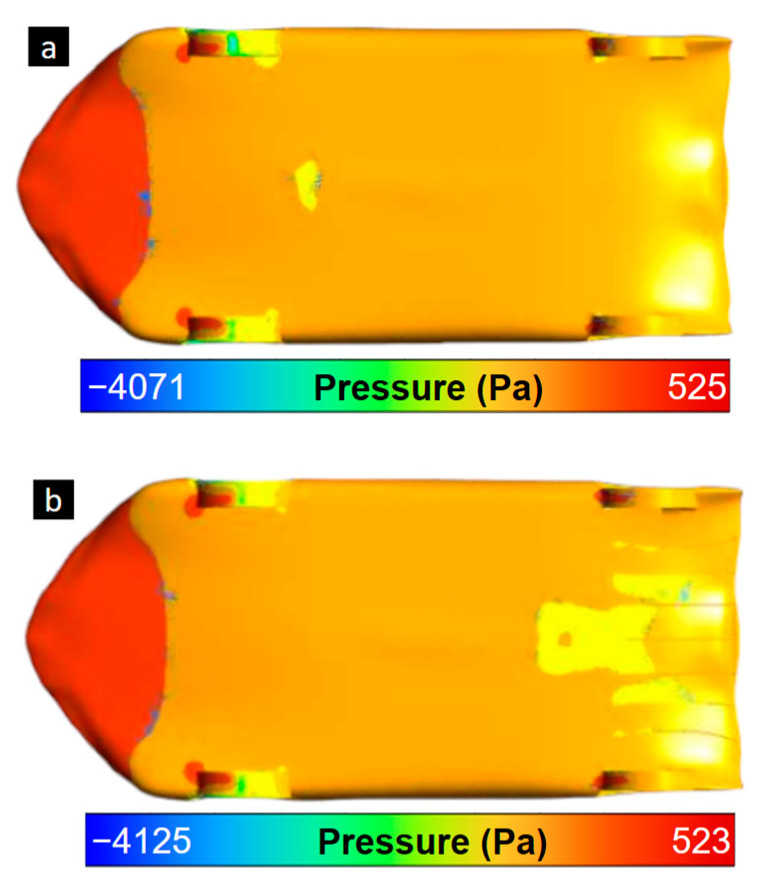
Pressure contour of Design 4 in the rear lower areas for original model (**a**) with no thin diffusers, and (**b**) with thin diffusers.

To observe the pressure difference generated by the aerodynamic diffusers, numerical results of the pressure contours are shown (Figure 16). The pressure variations in the diffuser area of Figure 16b are produced by the channeled air between the thin diffusers and the targeted increase in flow speed. This targeted effect was reached and produced an additional force towards the ground in the rear axle area for additional tire traction. The lift force was reduced by 40%, and the drag force showed minimal variation in its increase.

Since the diffusers clearly change the pressure distribution of the lower surface of the vehicle, slight variations to the pitch angle are expected, modifying the stability or maneuverability of the car. In the analyzed case, the higher pressure in the front of the lower surface and the reduction obtained in the rear part near the diffusers will increase the pitching moment of the car, affecting the computed downforce. Note that this pitch variation was not included in the present study; however, this effect could change the simulation results of velocity and pressure, streamline airflows and car stability. According to Szudarek et al. [47], generating aerodynamic downforce for the wheels on the front axle of a car is a much more difficult task than carrying this out for the rear axle. For this issue, using an inflatable splitter could yield sufficient low ground clearance, increasing the front axle’s downforce without a significant increase in the drag force [47]. Finally, dynamic analysis simulations and interactions with aerodynamics in the transient time domain could also be of interest; however, an integrated structural dynamics finite element method with computational fluid dynamics is required.

## 5. Discussion and Benchmarking of Numerical Results

The aerodynamic behavior of concept vehicle designs for underground conditions included several relevant analyzed parameters. First, the aerodynamic lift and drag coefficient of the four presented design are shown in Table 2. The drag forces decreased with the progression of the iterative process of different design configurations. The modifications to the vehicle’s front and rear areas showed the greatest impact on the results, with a reduction of 37% in the drag force from the initial design, Design 1, to the final design, Design 4 with diffusers. This reduction directly contributed to the vehicle’s efficiency and to the engine power required.

Even though the aerodynamic efficiency of the vehicle primarily involves reducing drag forces, effectively utilizing aerodynamics is crucial for achieving an efficient design. Lifting forces contribute to the stability and safety of the vehicle by directing aerodynamic loads towards the ground, improving adhesion to the pavement. During the design phase of the iterative process, modifications to the roof and underbody areas resulted in a 65% reduction in lift forces compared with those in the original design. This was achieved by taking into account that when the car is closer to the ground, faster wind flow underneath results in reduced pressure and load. Subsequently, the lift coefficient was optimized, achieving a reduced value of 0.05. The optimization process was performed by implementing the aerodynamic diffuser in the lower rear of the vehicle, resulting in an additional 58% lift coefficient reduction.

Note that variation in the aerodynamic drag coefficient (Cd) in real-life operations occurs depending on the vehicle accessories that affect the interaction of the external surface with the air flow [43]. Cooling has also been reported as the most influential factor, with a 10% increase in Cd [48,49]. Prediction errors of aerodynamic coefficients with computational fluid dynamics are reported by Zhang et al. [46] using a 1:3 scale sedan model in a wind tunnel. The remarkable reported numerical prediction accuracy of 99.35% indicates that this computational tool provides reliable and accurate results. In our case, to validate our numerical results and ensure their accuracy and reliability, the obtained drag coefficient was compared with the value ranges reported in the literature. The Mercedes-Benz prototype of the boxfish-inspired car, one of the most efficient models, was reported to have a Cd value of 0.19. The electric vehicle “Lucid Air” shows a Cd value of 0.21 [50]. The numerical Cd value of 0.26 was reported for a sedan with smoothed and simplified surfaces under similar conditions [46]. Others studies reported that thew Holden car had a Cd value of 0.4 at 100 km/h and of 0.44 with a taxi sign [43], and another boxfish-inspired car showed a Cd value of 0.24 [12]. Finally, we conclude that the validated model’s Cd value of 0.28 in our design is in close agreement with the range of other similar tested and simulated vehicles, including the well-known Ahmed benchmark body for external aerodynamics [40].

## 6. Conclusions

The aerodynamically efficient biomimetic vehicle design inspired by the mako shark shape owing to its exceptional operational kinetic qualities was developed and optimized using computational fluid dynamics modeling for underground tunnel conditions. The unique surface shape of the shark enabled remarkable optimized vehicle performance.

- The biomimetics-based vehicle design inspired by the mako shark achieved a drag coefficient (Cd) of 0.28.
- For the lift coefficient (Cl), the optimal design initially yielded a value of 0.055. Subsequent design enhancement using aerodynamic diffusers showed a pressure drop in the rear zone, increasing the downforce via a 58% reduction in the Cl. The resulting Cl value was 0.02.

This proposed biomimetic design process, coupled with computational fluid modeling, showed promising results for reducing the time and resources needed to design new car models with low aerodynamic drag for efficient automotive solutions towards a future with a low-carbon footprint.

Further work should focus on three main areas: first, adequate material selection for the development of a cost-effective concept model solution; second, the evaluation of the load-carrying capacity and energy impact absorptions of parts using advanced numerical modeling and simulations [51,52]; and third, an exploration of design optimization for lightweight vehicles through biomimetic and 3D-printing technology [53,54].

## Figures and Tables

**Table 1:** Numerical results of Design 4 with and without diffusers.

	Cd	Cl
Design 4	0.280	0.055
Design 4 with diffuser	0.286	0.023

**Table 2:** Aerodynamic coefficients of the four different designs.

	Design 1	Design 2	Design 3	Design 4	Design 4 with Diffuser
Cd	0.443	0.392	0.341	0.280	0.286
Cl	0.156	0.088	0.032	0.055	0.023

## Data Availability

The supporting information can be available from the corresponding author upon reasonable request.
